# 

**DOI:** 10.1017/S0144686X18000867

**Published:** 2018-10

**Authors:** CARWYN COOPER

**Affiliations:** Institute of Medical and Biomedical Education St George's, University of London, UK

This new book by Tony Walter is a pithy, sociological exploration of death, dying and bereavement in the modern world. The author has been researching, teaching and writing about these topics for many decades and this book aims to bring together some key insights from this body of work. The book scampers through a whole range of different topics, from the function of funerals to deaths in a digital world, and the breadth of issues covered is impressive. Somewhat inevitably, given the brevity of the book and the number of topics explored within it, some depth is sacrificed for breadth. However, this is not simply an introductory book. In each chapter the author quickly engages with cutting-edge research and contemporary controversies and he also casts a critical eye on the issues that he explores. This is especially true in the chapters discussing the ways in which people mourn and the value of talking about death. Indeed, the critique of the latter issue is refreshing, recognising as it does the fact that although talking about our mortality is beneficial for many, it is not something that everyone should either be required to do or criticised for not doing. The concluding chapter, which identifies a new paradigm called ‘pervasive death’, also takes the reader beyond the introductory and raises a number of difficult and novel challenges.

It is a little difficult to be sure at whom this book is targeted. The fact that it is so succinct and covers a great deal of ground, combined with the fact that each chapter finishes with a number of questions that look like they could be used as formative assessment essay questions, suggests that the primary audience is students or trainees; and I have no doubt that this book will be of use to these groups. However, although the book is relatively accessible and would clearly be helpful to those who need to learn about dying and grieving for an academic or professional purpose, there is plenty within it that should be of interest to more experienced researchers and practitioners. In particular, the discussion about social media and the impact of the digital on death in the modern world is revealing. Many of us now use social media on a regular basis and much work needs to be done to determine what should happen to our digital existences when our physical lives come to end. Few of us have given serious thought to this issue and yet it is clearly a complex matter that needs urgent attention.

The key weakness of this book lies in the decision by the author to deliberately exclude a number of important issues that are central to the narrative about death and dying in the 21st century. I have some sympathy with the decision to exclude some of these topics given that it would have been impossible to include them all in a short book. But not to discuss such controversial and contemporary issues as assisted suicide and euthanasia is remiss. This is especially true given that a number of jurisdictions, including Canada, have recently changed the legal landscape in this area. It was also an error not to discuss deaths in low- and middle-income countries. This omission greatly reduces the global relevance of the book for two reasons. Firstly, because deaths in low- and middle-income countries ‘comprise the majority of deaths today’ (p. 4) and, secondly, because most challenging death-related issues of all are currently encountered in these countries. In particular, the dire lack of palliative care provision in many low-income countries is a global scandal and needs to be brought to the attention of all students, researchers and practitioners.

To be fair, the book is not especially Eurocentric and there are plenty of references to different cultural practices in countries like Japan, New Zealand and the United States of America. Indeed, some of the most illuminating aspects of the book stem from the non-European examples that the author mentions in passing. For example, the brief discussion about a Japanese rite for aborted foetuses is fascinating. However, looking beyond the wealthier parts of our world would have been a real boon and would have given this book greater relevance to a global audience.

